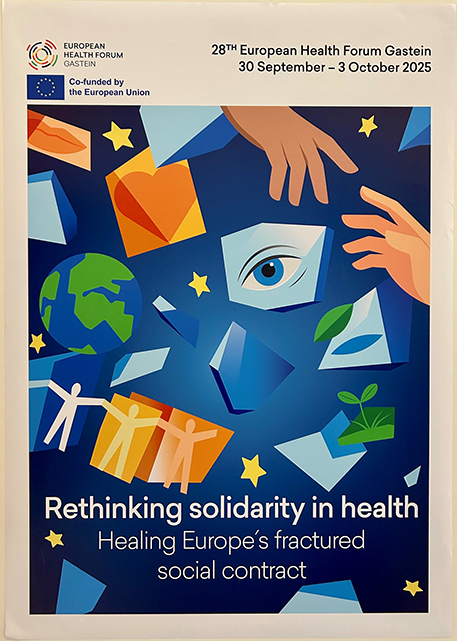# European Health Forum Gastein (EHFG) 2025

**DOI:** 10.1016/j.lanepe.2025.101521

**Published:** 2025-10-27

**Authors:** Ivana Nedic, Rebekka Park

The European Health Forum Gastein (EHFG) 2025, held in Bad Hofgastein, Austria, from 30 September to 3 October, brought together policymakers, health experts, and civil society leaders under the theme “Rethinking Solidarity in Health: Healing Europe's Fractured Social Contract.” The forum underscored a critical moment for European healthcare systems, highlighting mounting pressures and emerging solutions. Ivana Nedic and Rebekka Park highlight some key insights from the meeting.

## Rethinking solidarity in health

The first plenary, titled after this year's theme, “Rethinking Solidarity in Health,” set an urgent tone, with speakers warning that Europe's solidarity systems face unprecedented pressure from rising defence budgets, climate transition costs, and AI-driven automation. EHFG President Clemens Martin Auer described this as a pivotal moment and called for renewed social consciousness to safeguard the principles of solidarity and democracy that underpin the European way of life. Sandra Gallina, Director-General for Health and Food Safety at the European Commission, stressed the urgency of strengthening access, affordability, and fairness in healthcare, while Pamela Rendi-Wagner, Director of the European Centre for Disease Prevention and Control (ECDC), urged optimism and shared responsibility. The consensus emerged that rebuilding trust between institutions and citizens is essential to sustaining solidarity. The speakers emphasised that health depends not only on care systems but also on broader social and economic policies, requiring coordinated, cross-sectoral action to protect equity.

## The European Health Union

In the session “The European Health Union—Stronger together with networks in action” experts from various EU networks explored tools that strengthen preparedness for and prevention against health threats. In her introductory remarks, Sandra Gallina reaffirmed the establishment of a new network initiative, the EU Cardiovascular Health Plan, modelled on Europe's Beating Cancer Plan and focused on prevention, early detection, and treatment of Europe's leading cause of death. The full plan will follow later this year. The Medicines Shortages Steering Group, which monitors and predicts shortages, underlined the forthcoming Critical Medicines Act, expanding its remit with a procurement mandate to proactively coordinate and manage critical shortages.

## Health for people in vulnerable situations

The workshop on “Health for people in vulnerable situations” discussed solutions to address inequality within the European social pillar. The discussions built on findings from the latest report “Social inequalities in health in the EU” written by EuroHealthNet in collaboration with the Centre for Health Equity Analytics. The report found that nearly one-third of Europeans report less than good health and that inequalities between socioeconomic groups continue to widen. Thomas Maribo, Professor of Rehabilitation from Aarhus University, explained how socially differentiated interventions move away from diagnosis-focused care toward person-centred models that consider social context. Kristine Sørensen from the International Health Literacy Association emphasised not just the importance of individual health literacy, but system health literacy, where systems are designed to listen to and accommodate those at risk. Freek Spinnewijn, Director of the European Federation of National Organisations Working with Homeless People, encouraged the audience to expand understanding of social vulnerability and marginalisation. He noted that major EU health initiatives currently exclude homeless people. Insights from the panel will inform the new Action Plan for the European Pillar of Social Rights.

## A new era for geopolitics

The plenary titled “A New Era for Geopolitics—What Implications for a More Ambitious European Health Union?” explored how shifting global dynamics are reshaping health policy. Speakers noted that Europe faces both risks and opportunities as multilateral cooperation weakens and global norms evolve. European Commissioner for Health and Food Safety Olivér Várhelyi stressed that a healthy population underpins competitiveness and urged a shift from reactive to preventive policies. Panellists, including Ilona Kickbusch and Natasha Azzopardi Muscat, outlined priorities for the Health Union—from equity and strategic clarity to stronger global health leadership. Sanja Šišović of the International Youth Health Organisation emphasised youth engagement and intergenerational solidarity, highlighting the effects of conflict, climate change, and institutional distrust on young people's health and wellbeing. The plenary concluded that Europe must be more strategic and inclusive in global health for a fairer future.

## End of life, end of autonomy?

The session on “End of Life, End of Autonomy? Examining the Reality of the Current End-of-Life Paradigm” examined how end-of-life care reflects the social contract, with public services central to dignity and equity. Giovanna Marsico, Director of Le Centre National pour la Fin de Vie et les Soins Palliatifs, emphasised that decisions on end-of-life care require democratic debate, as access should not depend on income, postcode, or diagnosis. She added that policy debates often overlook social, cultural, and spiritual needs. The session underscored the tension between individual autonomy in dying and societal, ethical, and medical imperatives to preserve life. Dheepa Rajan of the European Observatory on Health Systems and Policies stressed that the debate must extend beyond medical professionals. The session concluded that sustaining dignity and quality of life requires stronger partnerships, inclusive policies, and recognition of both medical and non-medical needs.

The sessions highlighted here are a glimpse of what was discussed at EHFG 2025, and we encourage readers to explore the recordings of this meeting. Europe's political and economic context is undergoing major shifts that place countries under severe budgetary pressure, affecting health systems in unprecedented ways. The discussions at EHFG made it clear though that health equity and social justice must remain non-negotiables as stakeholders across all sectors rethink solidarity in health.

**Figure:**